# Dual role of melatonin as an anti-colitis and anti-extra intestinal alterations against acetic acid-induced colitis model in rats

**DOI:** 10.1038/s41598-022-10400-y

**Published:** 2022-04-15

**Authors:** Osama Ahmed, Alyaa Farid, Azza Elamir

**Affiliations:** Zoology Department, Faculty of Science, Cairo University, Giza, 12613 Egypt

**Keywords:** Gastrointestinal diseases, Immunological disorders, Experimental models of disease

## Abstract

The available ulcerative colitis drugs exhibit limited outcomes and adverse side effects. Therefore, our study aimed to investigate the therapeutic efficacy of melatonin in acetic acid (AA)-induced colitis to establish a possible treatment for colitis and its impacts on vital organs. Following colitis induction (2 ml 5% AA, rectally), rats were orally received melatonin (5 mg/kg) once per day for 6 days after colitis induction. Then, histopathological examination of colon, kidney, liver, and spleen was conducted, interleukin-1 beta (IL-1β), tumor necrosis factor-alpha (TNF-α), myeloperoxidase (MPO), malondialdehyde (MDA), glutathione (GSH), and total antioxidant capacity (TAC) levels were assessed in colon tissue. Colitis induction in untreated rats caused necrotic effects in colon tissues, a significant increase in colonic IL-1β, TNF-α, MPO, and MDA levels, and a remarkable decrease in GSH and TAC levels in colon tissue in comparison to the control group. Meanwhile, melatonin treatment reversed these parameters by improving the microscopic and macroscopic colitis features and extra-intestinal (kidney, liver, and spleen) changes in all treated rats compared to the colitis control group. These results denote a reduction in colitis severity due to the anti-inflammatory and anti-oxidative effects of melatonin and its positive impact on the vital organs.

## Introduction

Inflammatory bowel disease (IBD) is characterized by a sustained state of immune activation and inflammation within the gastrointestinal tract (GIT). The two most common inflammatory bowel disease phenotypes are Crohn's disease, which can affect any part of the GIT from the mouth to the anus, and ulcerative colitis, which affects the colonic and rectal mucosa^1,2^. The prevalence of colitis is increasing in developing nations, as well as in Egypt^3^. Even though the exact cause of IBD is still unidentified, environmental factors, infectious agents, immunological factors, and psychological factors, combined with genetic susceptibility, could all play a role in developing the disease^4^.

Several studies have shown that the GIT is a prominent site for creating reactive oxygen species (ROS), with the majority of ROS production attributed to microorganisms, dietary constituents, and interactions between immune cells^5,6^. Notably, acetic acid-induced colitis is a popular experimental model for ulcerative colitis because of its resemblance to human UC clinical symptoms and its simplicity and reproducibility. Acetic acid has the potential to break the epithelial barrier and disrupt the homeostasis between luminal antigens and intestinal immunity, which is the primary mechanism for the induction of colitis. Furthermore, increased ROS in IBD impair the total antioxidant capacity (TAC) of intestinal cells, resulting in a reduction in the entire antioxidant system of intestinal cells, including enzymatic and non-enzymatic antioxidants^7^. In the literature, it has been reported that ulcerative colitis is related to extra-intestinal alterations in essential organs such as the liver, spleen, and kidneys^8,9^. Increased intestinal permeability and inflammatory responses have been suggested as possible explanations for these changes^8,10,11^.

Currently, the primary treatments for IBD are sulfasalazine (Aminosalicylates), corticosteroids, immunosuppressive medications, and biological treatment^12^. On the other hand, these treatment approaches have limited good benefits and are associated with adverse side effects^13^. Recent research has revealed that the administration of antioxidants that also have anti-inflammatory properties, such as melatonin, may be beneficial in treating IBD^14^. The fact that melatonin is naturally produced in the GIT, in addition to the pineal gland, has been documented^15,16^. Melatonin has been revealed to have a variety of therapeutic properties, including antioxidant^17^, anti-inflammatory^18^, and immunoregulation^19^. In rat models of colitis, it was found to be effective in alleviating intestinal damage produced by dextran sulfate sodium^20^, also in a mice model^21^, dinitrobenzene sulfonic acid^22^, and trinitrobenzene sulfonic acid^23^.

This study aimed to investigate the effects of melatonin on histopathology of the colon, oxidative stress, and pro-inflammatory cytokines in acetic acid (AA)-induced colitis to establish a possible source for treating colitis. Furthermore, we aimed to assess whether melatonin could prevent the extra-intestinal alterations in vital organs by histological assessment of the liver, kidney, and spleen.

## Materials and methods

### Chemicals

Melatonin was purchased from Sigma Corporation. All other chemicals used were of analytical grade.

### Experimental animals and experimental design

Thirty-two male Albino Wistar rats (*Rattus norvegicus*) weighing 240–260 gm (6–9 weeks, age) were obtained from the National Research Center (NRC, Giza, Egypt). Animals were maintained in a friendly environment of a 12/12 h light–dark cycle at ambient room temperature (22–24 °C) in the animal house of the Department of Zoology, Faculty of Science, Cairo University, Egypt. Animals were fed standard chow and water ad libitum and kept for one week before any experimentation for acclimatization. Rats were divided randomly into four groups (8 rats/group):

**Negative control**: rats that received intracolonic instillation of 0.9% normal saline only, **melatonin control**: rats that received 5 mg/kg melatonin orally after intracolonic instillation with 0.9% normal saline, **positive control**: rats that were left untreated after colitis was induced, and **colitis + melatonin** group rats that received (5 mg/kg melatonin) after colitis induction. Treatments started 3 h after colitis induction and continued for 6 consecutive days once per day. Melatonin dose was determined according to the previous study of Dong et al.^23^.

### Ethical consideration

All the experimental procedures were performed under the international guidelines for the care and use of laboratory animals. The experimental animal protocol was approved (Protocol Number CU I F 38 19) by the Institutional Animal Care and Use Committee of Cairo University (CUIACUC) (Giza, Egypt). Our study experiments were performed in accordance with with ARRIVE guidelines.

### Colitis induction

Colitis induction was conducted, according to Rachmilewitz et al.^24^. In summary, all animals were fasted for 8 h prior to induction, with access to water, and then anesthetized with sodium pentobarbital (50 mg/kg, i.p)^25^. After the fasting time, colitis was induced by injecting 2 ml AA (5% v/v) in 0.9% normal saline intra-colonally, which was infused using a soft pediatric catheter (with an outer diameter of 2 mm, size 6F). The catheter was placed through the rectum into the colon to a depth of 8 cm, and the rat was kept in a supine Trendelenburg position to prevent acid from exiting the colon. After 30 s, the fluid was removed. Prior to withdrawing the AA, 2 ml of air was pumped into the colon to ensure that the AA was dispersed evenly throughout the colon. After removing the acid, the rectum was washed in the same volume of normal saline 0.9%.

### Animal handling and sample collection

Rats' weights (gram) were recorded daily throughout the experiment for observing weight change. Three hours after the last dose on day six, rats were euthanized by an overdose of sodium pentobarbital. Colons, spleens, kidneys, and livers of rats were rapidly excised and perfused in an ice-cold saline solution to remove red blood cells and clots. Proximal 8 cm of the colon was photographed to evaluate the macroscopic damage.

### Assessment of pro-inflammatory and oxidative stress parameters

Half gram of each colon tissue was homogenized in 4.5 ml of 10 mmol cold Tris–HCl buffer (pH 7.4), then centrifugated at 3000 rpm at 4 °C for 10 min. The supernatant was transferred into aliquots and frozen at − 80 °C until used. Lowry's method^26^ was performed for total protein content determination using a kit from Bio Basic Inc. (Cat. SK4031, Canada).

#### Myeloperoxidase (MPO), malondialdehyde (MDA), reduced glutathione (GSH), and total antioxidant capacity (TAC) levels

Colonic MPO activity was determined using BioVision Inc. kit (Cat. E4581, CA, USA) according to the manufacturer's protocol. Colonic MDA was assessed spectrophotometrically using Ohkawa et al*.* method^27^ using a kit from BioVision Inc. (Cat. K739, CA, USA). According to the manufacturer's protocol, the colonic GSH was measured using a kit from BioVision (Cat. K264, CA, USA), and TAC levels in colonic tissue were evaluated using a kit from LSBio Inc. (Cat. LS-F14457).

#### Pro-inflammatory cytokine assays

The colonic pro-inflammatory cytokine, interleukin-1beta, was measured by Rat IL-1β kit (Cat. ab100768, Abcam, USA), while tumor necrosis factor-alpha was assessed by rat TNF-α kit purchased from Bio-legend Inc. (Cat. 438,206, USA). Sandwich enzyme-linked immunosorbent assay (ELISA) was performed according to manufacture instructions. Absorbances were detected using an ELISA plate reader at a wavelength of 450 nm.

### Histopathological examination

Organs of each rat (including Liver, Kidney, Spleen, and colon) were collected from all experimental groups at the end of the study, then fixed in 10% neutral buffered formalin, embedded in paraffin, sectioned into 4–5 µm sections, deparaffinized in xylene, hydrated and stained with hematoxylin and eosin (H&E), and then examined by light microscope by a pathologist who is unware of the experimental design. Histopathological scoring of colitis was done according to Ahmed et al.^14^; the damage score assigned for each section was from (0 to 3) for each of the five parameters, including **mucosal necrosis** (0: No necrosis, 1: Focal necrosis/erosion, 2: Partially necrotic/ulcerated, 3: Totally necrotic/ulcerated), **mucosal inflammatory cells infiltration** (0: No inflammatory cells, 1: Mild 2: Moderate, 3: Marked), **submucosal inflammatory cells infiltration** (0: No inflammatory cells, 1: Mild, 2: Moderate, 3: Marked), **fibrosis** (0: No fibrosis, 1: Mild, 2: Moderate, 3: Marked), and finally **submucosal edema** (0: No edema, 1: Mild, 2: Moderate, 3: Marked). The total histopathological score for each group colon was calculated by the mean of 5 parameters of 8 colon samples collected from each group.

### Statistical analysis

The IBM statistical package for the social sciences version 25 was used to analyze the data (copyright by IBM SPSS software, US). The Kolmogorov–Smirnov test was used to ensure that the data were normally distributed, then parametric statistical analysis was conducted. One-way analysis of variances (ANOVA) evaluated the changes between the experimental biomarkers. *P* < 0.05 was considered to be the minimal level of significance. A post hoc ANOVA (Tukey's homogeneity test) was employed to determine the differences and similarities between the experimental groups. The mean and standard error of the mean (SEM) were used to express all the results. GraphPad Prism version 5 was used to visualize the data (Graph Pad Software Inc., San Diego, CA). The improvement percent for each parameter was calculated according to the following equation:

$${\text{Improvement }}\% = \frac{Melatonin\, treated\;group - colitis\; untreated \;group}{{colitis \;untreated\; group}} \times 100$$

### Ethics approval and consent to participate

The experimental animal protocol was approved (Protocol Number CU I F 38 19) by the Institutional Animal Care and Use Committee of Cairo University (CUIACUC) (Giza, Egypt).

## Results

### Effect of melatonin on TAC and oxidative stress parameters (MPO, MDA, and GSH)

**Figure 1:**
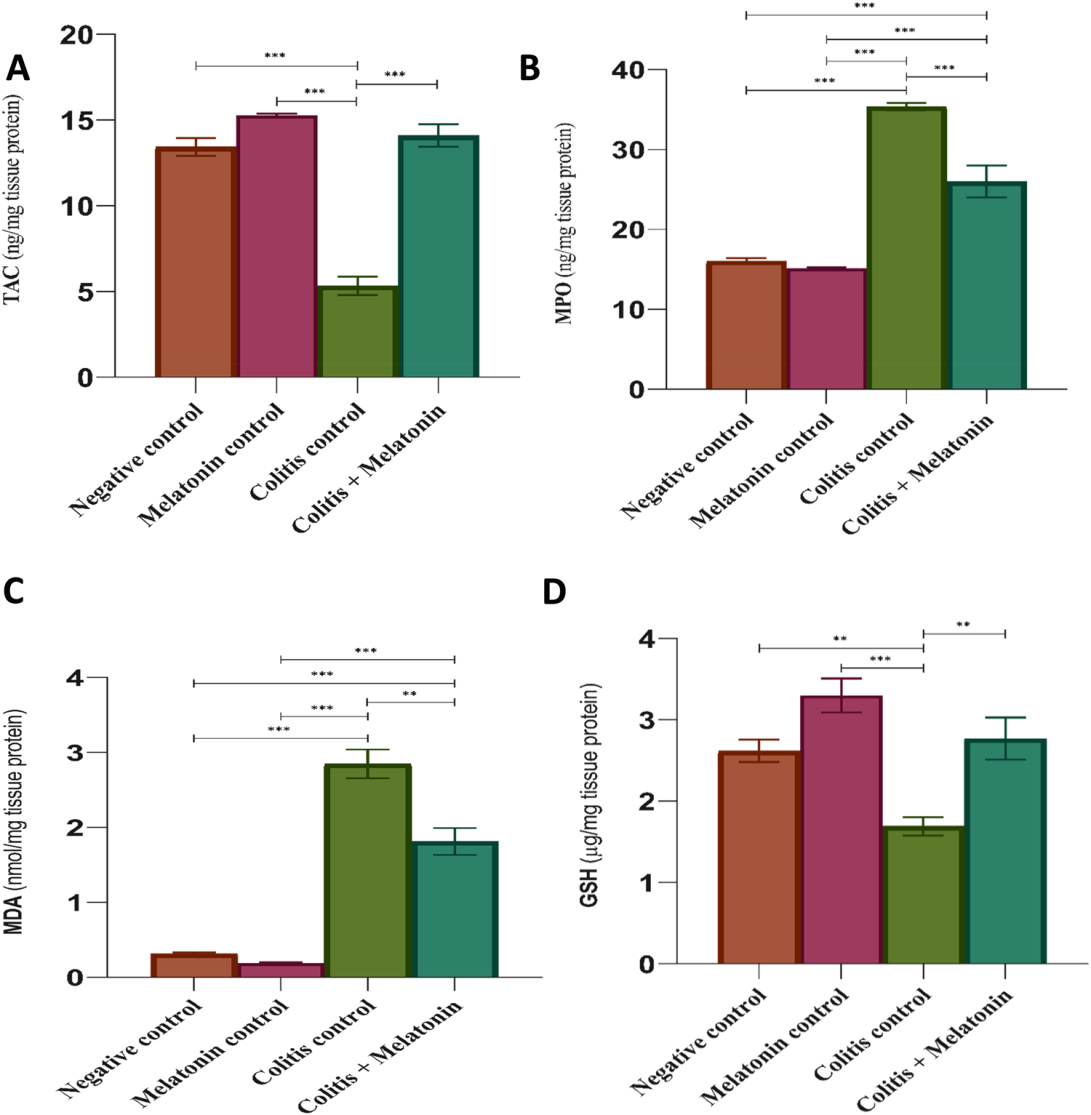
Effect of melatonin (5 mg/kg) on myeloperoxidase (MPO) level (**A**), colonic total antioxidant capacity (TAC) (**B**), colonic malondialdehyde (MDA) level (**C**), and glutathione reduced (GSH) level (D) levels after 6 days of colitis induction, values are expressed as mean ± SEM for 8 rats per group, **p < 0.001 and ***p < 0.0001 as determined by one-way ANOVA followed by Tukey's homogeneity test.

When compared to the negative control group, the colitis-induced rats had a significant decrease (*P* < 0.0001) in TAC levels, a significant increase (*P* < 0.0001) in MPO and MDA levels, and a significant decrease (*P* < 0.001) in GSH levels. Meanwhile, when comparing the colitis + melatonin (5 mg/kg) group to the colitis group without treatment, a significant decline in MPO (*P* < 0.0001) (− 26.44%) and MDA (*P* < 0.001) (− 36.38%) levels, as well as a significant increase (*P* < 0.001) (+ 63.78%) in GSH levels, were noted. In addition, the treatment with melatonin resulted in a statistically significant increase (*P* < 0.0001) (+ 164.16%) in TAC levels compared to the colitis-induced group that was not treated. In contrast, no significant change was observed between the melatonin-treated group after colitis induction with the negative and melatonin controls except for MPO and MDA, where a significant increase (*P* < 0.0001) was observed, as shown in Fig. 1A-D.

### Effect of melatonin on pro-inflammatory cytokines (IL-1β and TNF-α)

**Figure 2:**
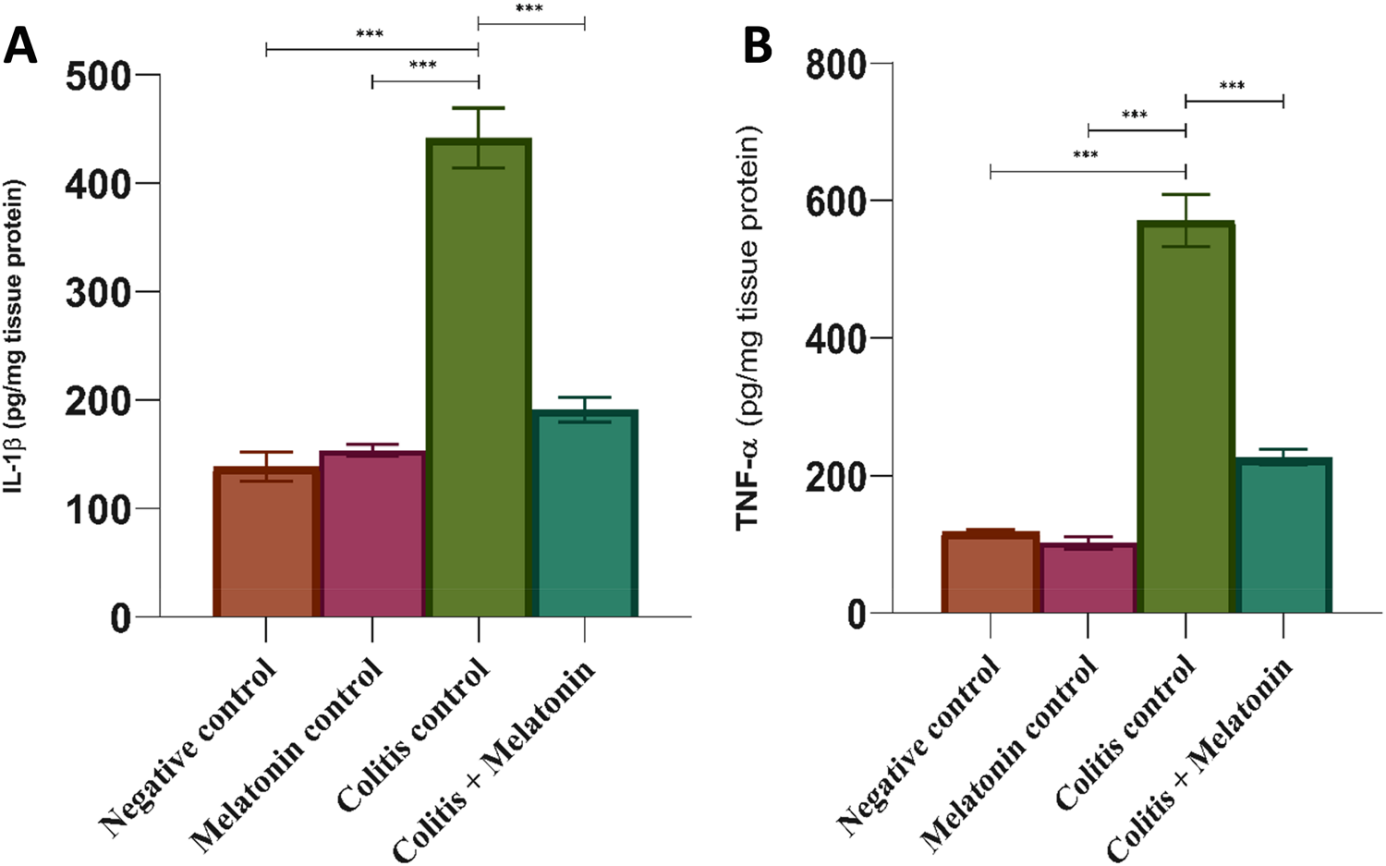
Anti-inflammatory Effect of melatonin (5 mg/kg) on colonic IL-1β (**A**) and TNF-α (**B**) levels after 6 days of colitis induction, values are expressed as mean ± SEM for 8 rats per group, ***p < 0.0001 as determined by one-way ANOVA followed by Tukey's homogeneity test.

Acetic acid-induced colitis in rats showed a significant increment (*P* < 0.0001) in IL-1β and TNF-α levels compared with the negative control group. Meanwhile, a significant decrease (*P* < 0.0001) in these parameters mentioned above was observed in the colitis + melatonin group (5 mg/kg, orally) (Improvement% for IL-1β -56.72%, for TNF-α -60.29%) compared with the colitis group without treatment. Besides, the treatment with melatonin had no significant change with the negative and melatonin control groups, as shown in Fig. 2A,B.

### Histopathological features of colon and weight change

**Figure 3:**
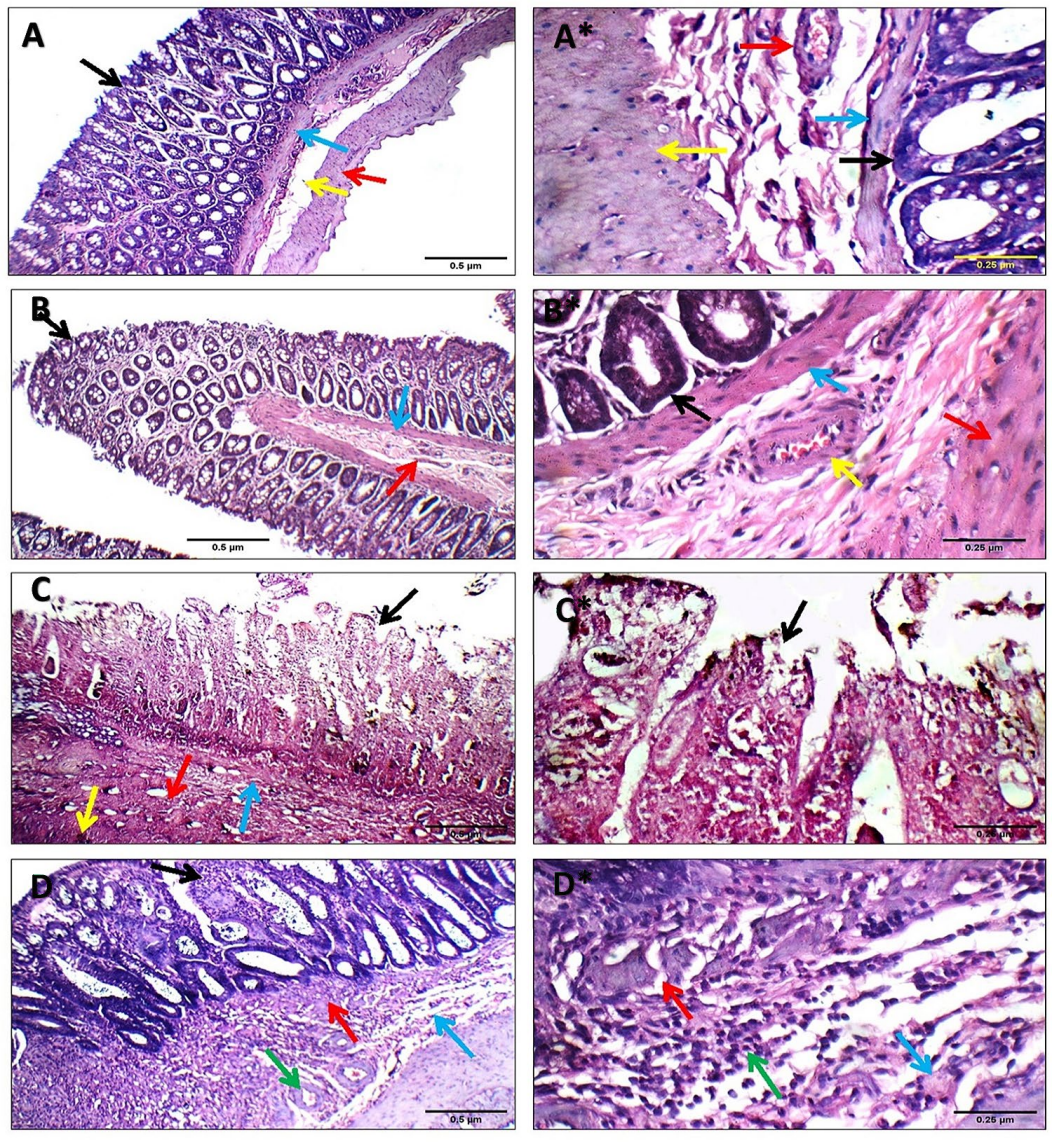
Effect of melatonin (5 mg/kg) on colon histology. (**A**) colon from the negative control group showing intact viable mucosa (black arrow), average muscularis mucosa (blue arrow), average submucosa (yellow arrow), and average musculosa (red arrow) (H&E X200), A* (H&E X400); (**B**) Melatonin control colon showing intact viable mucosa (black arrow), average muscularis mucosa (blue arrow), and average submucosa with average blood vessels (red arrow) (H&E X200), B* (H&E X400); (**C**) Colitis control colon showing totally necrotic mucosa (black arrow), muscularis mucosa (blue arrow), submucosa (red arrow), and musculosa (yellow arrow) (H&E X200), C* (H&E X400); (**D**) Colitis + melatonin; colonic wall showing mucosa with mild inflammatory infiltrate (black arrow), thickened muscularis mucosa (red arrow), and submucosa showing mild edema (blue arrow) with average blood vessels (green arrow) (H&E X200), D* (H&E X400).

Upon microscopic examination (H&E X200 and X400), the colonic wall of the negative control and melatonin control groups revealed that the colonic wall had intact viable mucosa and superficial layer, normal muscularis mucosa, submucosa, and musculosa, with no inflammatory cellular infiltrate, and no inflammatory cellular infiltrate (Fig. 3A,B). In comparison to the negative control group, after 6 days of colitis induction with single instillation of acetic acid caused significant (*P* < 0.01) necrotic effects on the mucosa, submucosa, and musculosa, with a prominent inflammatory infiltration and dilated congested blood vessels (Fig. 3C). Meanwhile, when compared to the colitis control group, the melatonin-treated colitis group had a lower colitis parameter than the colitis control group (Fig. 3D).

**Figure 4:**
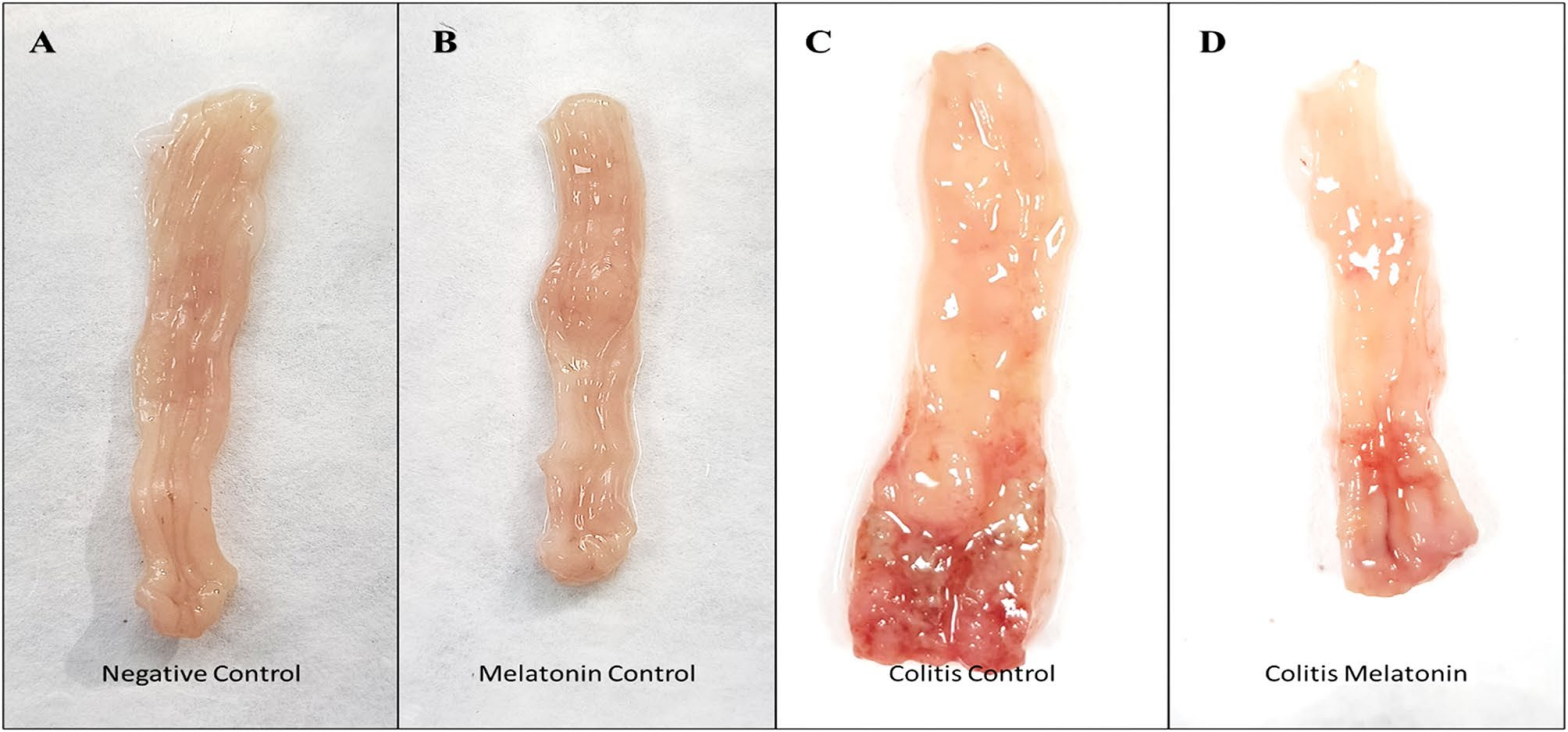
Effect of melatonin (5 mg/kg) on macroscopic colon appearance after 6 days of colitis induction with 5% acetic acid.

**Figure 5:**
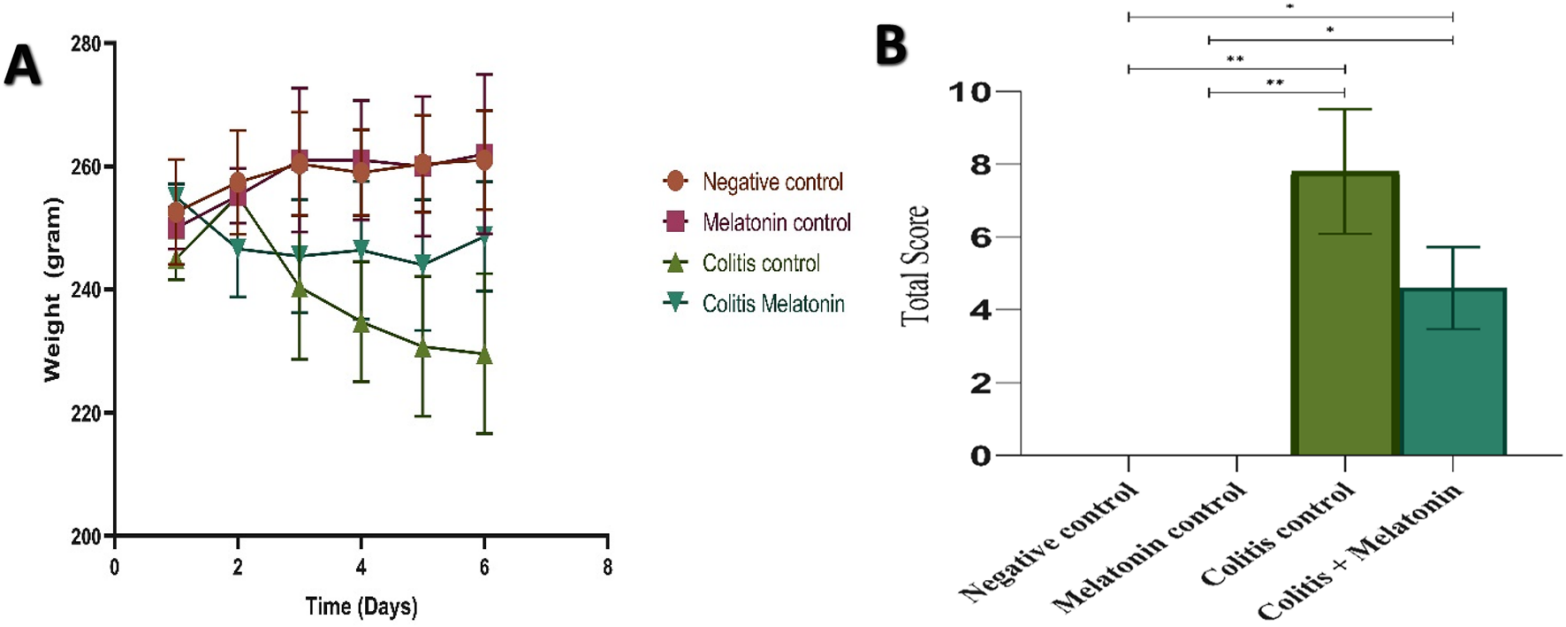
(**A**) Graph shows the bodyweight change in each group, (**B**) graph shows the total histopathological score index for colitis; The total histopathological score for each group colon was calculated by the mean of 5 parameters of 8 colon samples collected from each group, values are expressed as mean ± SEM for eight rats per group, *p < 0.05 and **p < 0.001 as determined by one-way ANOVA followed by Tukey's homogeneity test.

Bodyweight loss was reported following the instillation of acetic acid through the rectal route. The macroscopic features of the colons of the negative control and melatonin control groups were normal with no damage observed in the mucosa layer, while in the colitis control group, a severe edematous mucosal inflammation was observed; furthermore, this observation was reduced upon melatonin treatment in the melatonin treated group (Fig. 4A-D). The weights of the rats in both the negative control and the melatonin control groups increased over the treatment period while in the colitis intreated group, a weight loss was observed started from the second day till the end of the experiment, while this loss was inhibited in the melatonin treated group (Fig. 5A). The colitis treated with the melatonin group showed a lower score from the colitis control group -41.02% in terms of colitis severity, as presented in Fig. 5B.

### Histopathological features of kidney, liver, and spleen

**Figure 6:**
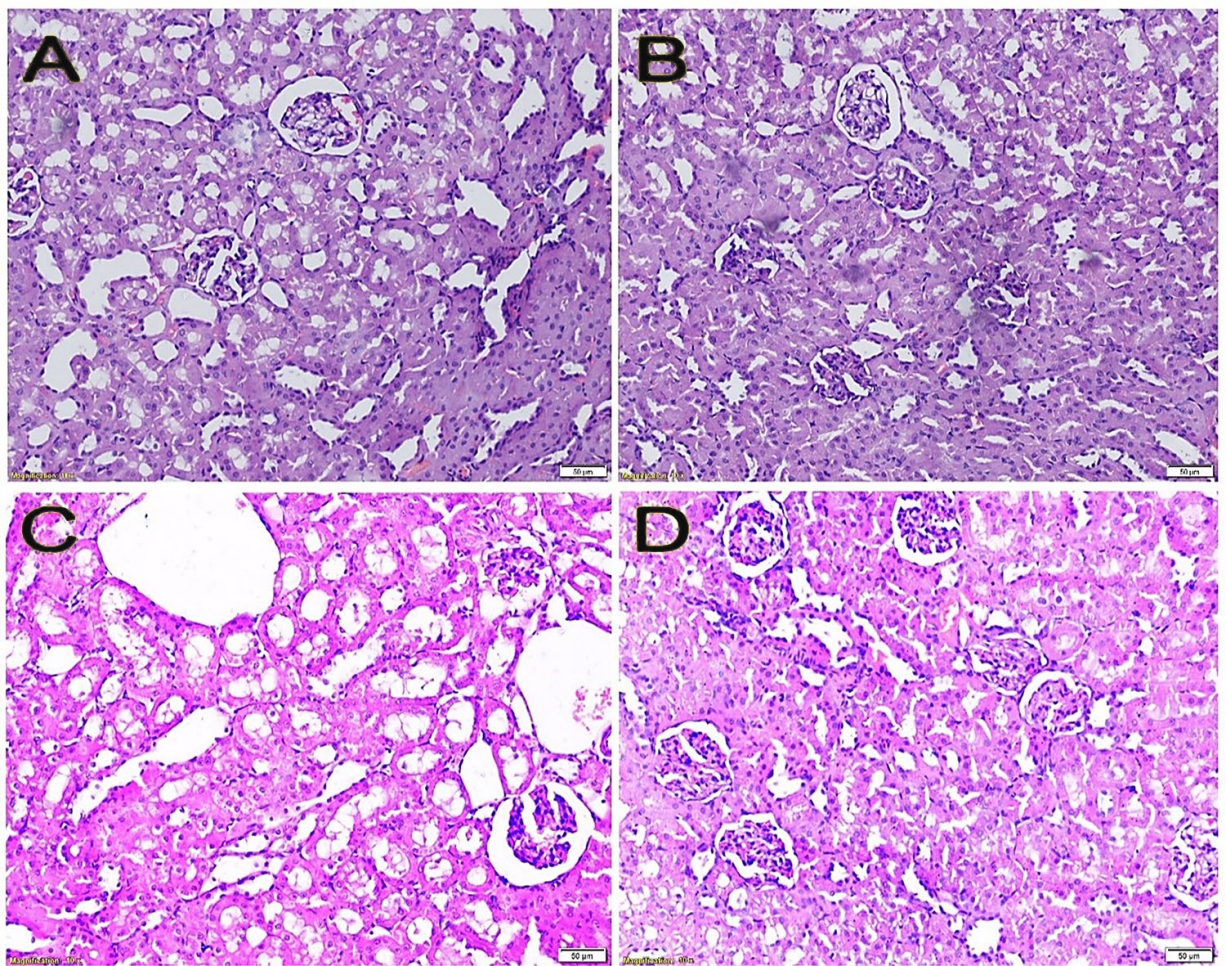
Kidney sections stained with hematoxylin and eosin (H&E) showing (**A**) average renal capsule, glomeruli, average tubules, and interstitium (negative control group), (**B**) average renal capsule, glomeruli, tubules, and interstitium (melatonin control group), (**C**) small-sized glomeruli with wide bowman's space, and dilated congested blood vessels, with areas of hemorrhage (colitis control group), (**D**) average glomeruli, tubules and interstitium (colitis + melatonin group).

**Figure 7:**
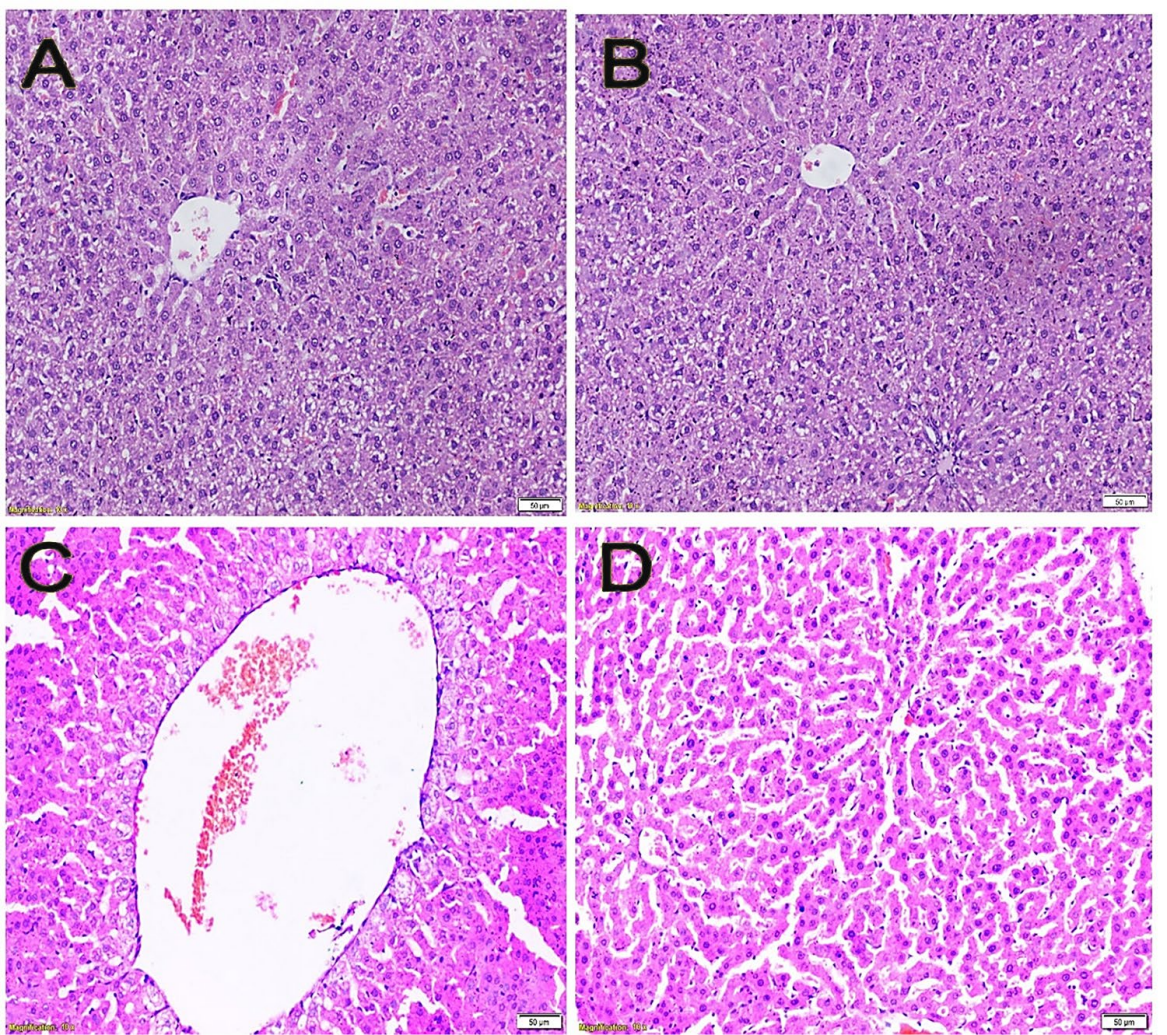
Liver sections stained with H&E showing (**A**) average central vein with regular hepatocyte strands (negative control), (**B**) average central veins with regular hepatocyte strands (melatonin control group), (**C**) marked dilated congested central vein and hydropic change of hepatocytes (colitis control group), (**D**) average central vein with regular hepatocyte strands (colitis + melatonin group).

**Figure 8:**
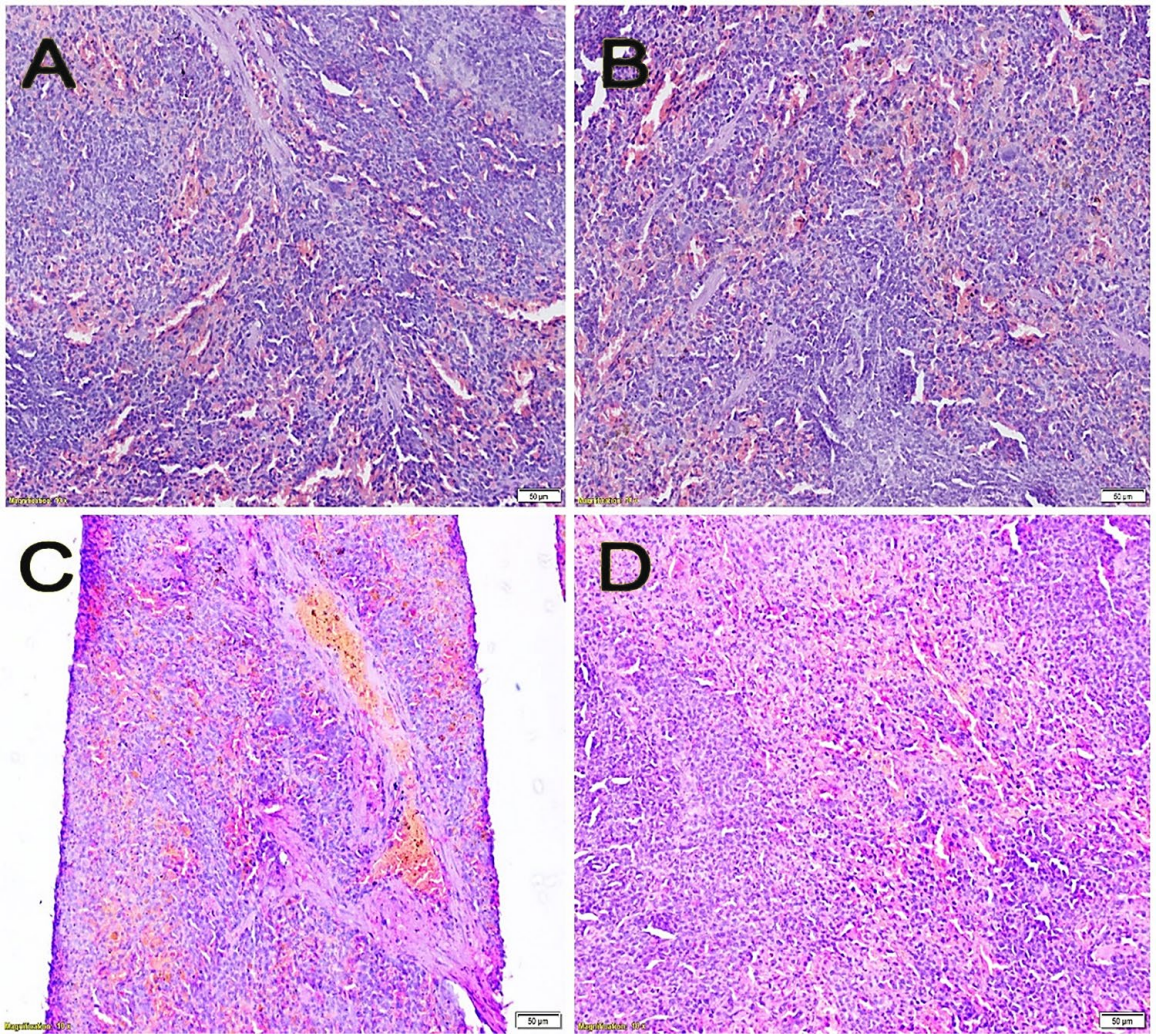
Spleen sections stained with H&E showing (**A**) average lymphoid follicles and average blood sinusoids (negative control), (**B**) average lymphoid follicles and average blood sinusoids (melatonin control group), (**C**) markedly congested blood sinusoids (colitis control group), (**D**) average lymphoid follicles and blood sinusoids (colitis + melatonin group).

The glomeruli, tubules, and interstitium in kidney sections from the negative control and melatonin control groups (Fig. 6A,B) were of average size and shape. It was observed that the colitis-induced group had histopathological changes such as dilated congested blood vessels with hemorrhage and small-sized glomeruli with wide bowman's space (Fig. 6C). In contrast, no significant abnormalities were noticed when examining colitis rats administered with melatonin (Fig. 6D). It was shown that the central vein of the colitis-induced group was dilated and congested, with a hydropic alteration in the hepatocytes (Fig. 7C). Sections of the spleen taken from the colitis-induced group revealed significantly congested blood sinusoids (Fig. 8C). The histological profile of the melatonin-treated group (in kidney, liver, and spleen) (Figs. 6D, 7D, 8D) was similar to that of the negative control and melatonin control groups (Figs. 6A,B, 7A,B, 8A,B).

## Discussion

Ulcerative colitis (UC) is an inflammatory bowel disease (IBD) that generates inflammation and ulcers in the mucosa of the colon^1,2^. Because of the adverse side effects and restricted outcomes of the traditional treatments being used, it is necessary to study new product lines with more desirable therapeutic profiles to improve the outcomes^13^.

According to the literature, several inflammatory responses in the inflamed mucosa have been shown to be modulated by ROS, stimulating the release of a wide range of inflammatory cytokines in a variety of tissues, hence exacerbating tissue damage. During oxidation, free radicals attack polyunsaturated fatty acids of the plasma membranes, causing their decomposition and deterioration. This mechanism is critical in the etiology of colitis^28^. Inflammatory markers in the colon involving MPO, an enzyme present in neutrophils, and MDA, one of the end products of lipid peroxidation; both have been employed as quantitative measures of colon inflammation^29,30^. GSH is a primary antioxidant agent that is crucial in the tissue repair process because it prevents free radicals from damaging the mucosa, which is essential for wound healing. During inflammation, GSH drops, leading to considerable colon mucosal deterioration. As a result, GSH is critical in protecting intestinal cells during instances of inflammation^31^. In our investigation, MPO and MDA levels were shown to be elevated in the colon tissue of the colitis control group, whereas GSH levels were found to be decreased. These observations come in parallel with prior investigations^14,32,33^. Moreover, the total antioxidant capacity (TAC) is reduced in both IBD phenotypes^7^. In our study, the TAC levels in the untreated colitis group were significantly lower compared to the negative control group, while a significant increment was observed in melatonin-treated rats. Furthermore, the observations of colon histology in the untreated colitis control group demonstrated an increase in inflammatory cells infiltration, leading to increased levels of oxidative stress. Also, ulcerative colitis is associated with extra-intestinal changes in vital organs such as the liver, spleen, and kidney; these alterations can be explained by the increased intestinal permeability and inflammatory reactions characteristic of ulcerative colitis. Moreover, our results explored that alterations in the histo-morphological structures of the liver, spleen, and kidney in the untreated colitis group, which is in agreement with previous studies^8–11,16^. Furthermore, the treatment with melatonin prevented these extra-intestinal alterations, as no histopathological changes were observed in these organs microscopically.

Inflammatory reactions and cytokine profiles, such as IL-1β and TNF-α, are abundantly produced and associated with pathological alterations in IBD^34^. These cytokines drive downstream immune responses^35–37^. During active ulcerative colitis, IL-1β, one of the most prominent mediators of colon inflammation, is secreted in greater quantities^36^. Furthermore, macrophages and lymphocytes secrete TNF-α, an exceptionally strong cytokine, during the onset of an inflammatory response^38^. Additionally, TNF-α stimulates the production of pro-inflammatory cytokines, including IL-1β^39^. According to the current study, AA instillation in rats with colitis generated statistically significant rises in levels of IL-1β and TNF-α, which is similar to prior research by Ghasemi et al.^40^. Due to increased epithelial permeability, luminal antigens penetrate the lamina propria layer and activate the mucosal immune system, resulting in an over-reaction of the mucosal immune system, which results in the excessive production of pro-inflammatory cytokines^41–45^.

When the AA-induced colitis group was compared to the negative control group, the microscopic examination of the colon revealed significant necrosis in the mucosa layer with significant inflammatory cell infiltration in the AA-induced colitis group, which was not observed in the negative control group. According to the findings of Tahan et al.^46^, Rectal instillation with AA could cause severe mucosal injury, infiltration of pro-inflammatory cells, and ulceration. The increase in MPO, MDA, IL-1β, and TNF-α, as well as the decrease in GSH and TAC, were all observed in our study, supporting the microscopic examination.

Interestingly, the inhibition of IL-1β and TNF-α has been found to reduce colitis^47–49^. Remarkably, melatonin administration lowered IL-1β and TNF-α levels, which might be reasoned by the anti-inflammatory impact of melatonin on the inflamed colon. Our observation corresponds with a prior study^50^, where melatonin supplementation suppressed pro-inflammatory cytokines, including IL-1β and TNF-α, in the DSS-colitis model in rats. Also, treatment with melatonin induced a remarkable reduction in colitis histopathology's severity compared to the colitis control group. This impact is presumably attributable to the anti-inflammatory and antioxidant characteristics of melatonin^51–53^. Its impact on colitis was evidenced by improving the biochemical markers TAC and GSH and the decrease in IL-1β, TNF-α, MDA, and MPO compared to the colitis control group. In this context, neutrophil infiltration can be stimulated by TNF-α production^38^. In our investigation, neutrophil infiltration and TNF-α level decreased in the colon after treatments with melatonin. Also, the treatment with melatonin was found to augment and maintain the vital organs, including the liver, kidney, and spleen. Supporting that melatonin treatment has an anti-inflammatory and antioxidant effect on colons and the vital organs, as evidenced in the microscopic examination of these organs in the colitis treated with melatonin group compared to the untreated colitis group.

In conclusion, this study suggests that treatment with melatonin may be beneficial in the prevention of experimental colitis in rats due to its antioxidant and anti-inflammatory properties, which were observed in the study in reducing MPO and MDA levels, pro-inflammatory cytokines (IL-1β and TNF-α), increasing GSH activity, and total antioxidant capacity.

## Data Availability

The datasets used and/or analysed during the current study available from the corresponding author on reasonable request.
